# Molecular typing of *Mycobacterium tuberculosis*: a review of current methods, databases, softwares, and analytical tools

**DOI:** 10.1093/femsre/fuaf017

**Published:** 2025-04-26

**Authors:** David Couvin, Anne-Sophie Allaguy, Ayoub Ez-zari, Tomasz Jagielski, Nalin Rastogi

**Affiliations:** WHO Supranational TB Reference Laboratory—TB and Mycobacteria Unit, Institut Pasteur de la Guadeloupe, F-97139, Les Abymes, Guadeloupe, France; Laboratoire de Mathématiques Informatique et Applications (LAMIA), Université des Antilles, F-97154, Pointe-à-Pitre, Guadeloupe, France; Laboratoire de Mathématiques Informatique et Applications (LAMIA), Université des Antilles, F-97154, Pointe-à-Pitre, Guadeloupe, France; Laboratory of Biology and Health (UAE/U06FS), Department of Biology, Faculty of Science, Abdelmalek Essaâdi University, BP 2121, 93002 Tetouan, Morocco; Department of Medical Microbiology, Institute of Microbiology, Faculty of Biology, University of Warsaw, I. Miecznikowa 1, 02-096 Warsaw, Poland; WHO Supranational TB Reference Laboratory—TB and Mycobacteria Unit, Institut Pasteur de la Guadeloupe, F-97139, Les Abymes, Guadeloupe, France

**Keywords:** *Mycobacterium tuberculosis*, tuberculosis, epidemiology, software, database, drug resistance, family, genomics, genotyping, lineage

## Abstract

Studies on the epidemiology and clinical relevance of *Mycobacterium tuberculosis* complex (MTBC) have immensely benefited from molecular typing methods, associated software applications, and bioinformatics tools. Over the last two decades, the Pasteur Institute of Guadeloupe has developed a range of bioinformatic resources, including databases and software, to advance understanding of TB epidemiology. Traditional methods, such as IS*6110*-RFLP, MIRU-VNTR typing, and spoligotyping, have been instrumental but are increasingly supplanted by more precise and high-throughput techniques. These typing methods offer relatively good discrimination and reproducibility, making them popular choices for epidemiological studies. However, the advent of whole-genome sequencing (WGS) has revolutionized *Mycobacterium tuberculosis* complex (MTBC) typing, providing unparalleled resolution and data analysis depth. WGS enables the identification of single nucleotide polymorphisms and other genetic variations, facilitating robust phylogenetic reconstructions, and detailed outbreak investigations. This review summarizes current molecular typing methods, as well as databases and software tools used for MTBC data analysis. A comprehensive comparison of available tools and databases is provided to guide future research on the epidemiology of TB and pathogen-associated variables (drug resistance or virulence) and public health initiatives.

## Teaser

This article reviews molecular typing techniques, with a particular focus on whole-genome sequencing and its transformative role in the research of tuberculosis.

## Introduction

Tuberculosis (TB), caused by *Mycobacterium tuberculosis* complex (MTBC), remains a critical global health issue. According to the World Health Organization (WHO) Global TB Report 2024, 10.8 million people developed TB in 2023, and 1.25 million people succumbed to the disease. Encouragingly, 74 million lives have been saved since 2000 through WHO-guided worldwide TB control actions. TB is widespread throughout the world, yet it disproportionately affects low-income regions, including Sub-Saharan Africa and South Asia, in particular 30 high TB burden countries (HBCs), which accounted for 87% of the global TB burden in 2023. Limited healthcare infrastructure certainly exacerbates the epidemic in HBCs, since the disease has been linked to poverty, malnutrition, promiscuity, poor living conditions, and other unfavorable socio-demographic factors (Farmer et al. 2006, Keshavjee et al. 2008). Furthermore, HIV infection and drug resistance, whose prevalence has been particularly high in many HBCs, are major impediments to successful TB treatment, and have considerably contributed to the persistence of the global TB epidemic. Drug-resistant *Mycobacterium tuberculosis* has recently been introduced into the WHO bacterial priority pathogens list, 2024 (https://www.who.int/publications/i/item/9789240093461), further emphasizing the need to improve TB control and prevention, especially in resource-limited settings.

**Figure 1.**
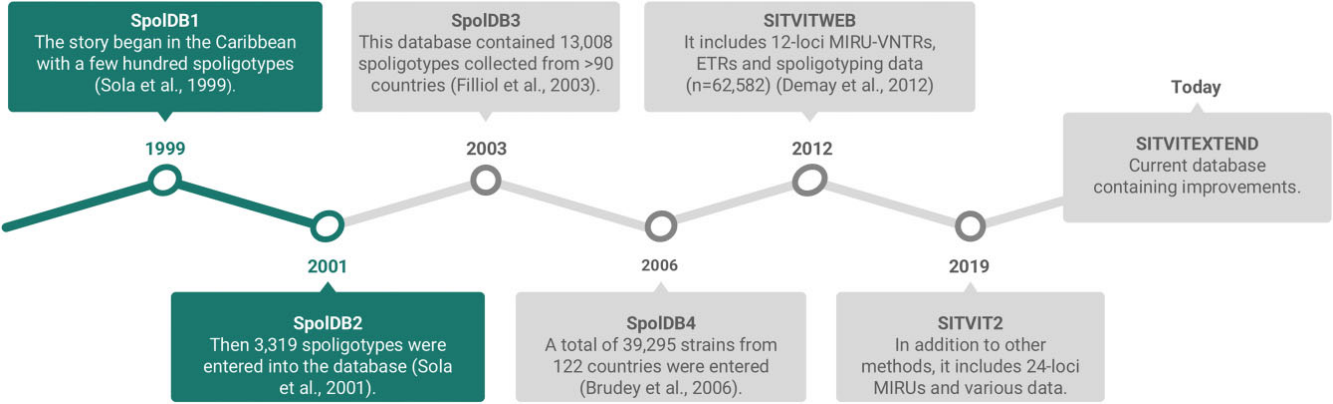
Brief histogram of SpolDB/SITVIT database evolution.

The MTBC includes a group of closely related species (e.g. *Mycobacterium tuberculosis sensu stricto, Mycobacterium africanum, Mycobacterium bovis, Mycobacterium caprae, Mycobacterium pinnipedii, Mycobacterium suricattae, Mycobacterium orygis, Mycobacterium microti, Mycobacterium mungi*, and probably other species) that are potentially pathogenic for both humans and animals. These species generally belong to one or more phylogenetic lineages (or clades/families). TB-dedicated genotyping databases and software tools developed in the Institut Pasteur de la Guadeloupe are able to provide a holistic view of specific aspects of TB research by aiding the analysis of data collected from numerous TB laboratories worldwide (data available for >128 000 MTBC strains from 160 countries; the most recent versions under development being SITVITEXTEND and SITVITGeno). Figure 1 displays a brief history of evolution of various SpolDB/SITVIT databases over time in our laboratory (Sola et al. 1999, 2001, Filliol et al. 2003, Brudey et al. 2006, Demay et al. 2012, Couvin et al. 2019, 2022).

In this manuscript, we review several molecular tools, such as IS*6110*-RFLP, spoligotyping, MIRU-VNTR, and whole-genome sequencing (WGS), that have revolutionized TB epidemiology, offering insights into genetic diversity, transmission patterns, and drug resistance mechanisms. This review evaluates current molecular typing methods, WGS advancements, and bioinformatics tools, emphasizing their potential to address critical gaps such as inadequate strain characterization, limited access to drug resistance data, and challenges in tracing transmission dynamics in TB-endemic regions.

## Chapter 1: an overview of TB molecular typing techniques

Since the early understanding of TB transmissibility and control, monitoring and surveillance of the disease have traditionally relied on conventional methods, such as contact tracing, which involve human interviews and the collection and analysis of extensive demographic and clinical data (Fox et al. 2013). Over the past three decades, the emergence of molecular epidemiology has significantly enhanced our understanding of TB transmission and evolution, thereby greatly contributing to both public health strategies and clinical management of this devastating disease (Jagielski et al. 2016).

Molecular typing, or genotyping, of *M. tuberculosis* strains is a cornerstone of molecular TB epidemiology. A fundamental assumption is that strains with identical or highly similar genotyping patterns form a “genotypic cluster,” which is considered a proxy for cases arising from recent transmission. Despite variations in the criteria used to define genetic identity, genotyping has proven irreplaceable in investigating outbreaks, deciphering chains of transmission or distinguishing between relapses and reinfections.

Furthermore, molecular typing has been crucial in phylogenetic and evolutionary studies of TB, allowing to identify major lineages and emerging clones, which are often associated with specific geographical regions, increased transmission rates, or acquisition of virulence and drug resistance traits (Mathema et al. 2006, Manson et al. 2017, Dookie et al. 2018). Overall, genotyping has become an essential tool for gaining deep insights into the genetic diversity, prevalence patterns, circulation, and evolution of the pathogen.

Both historical and contemporary methods of *M. tuberculosis* genotyping were reviewed earlier (Mathema et al. 2006, Jagielski et al. 2014, 2016). Here, we briefly describe and update the most widely accepted and currently used modalities.

### IS*6110*-RFLP typing

**Figure 2.**
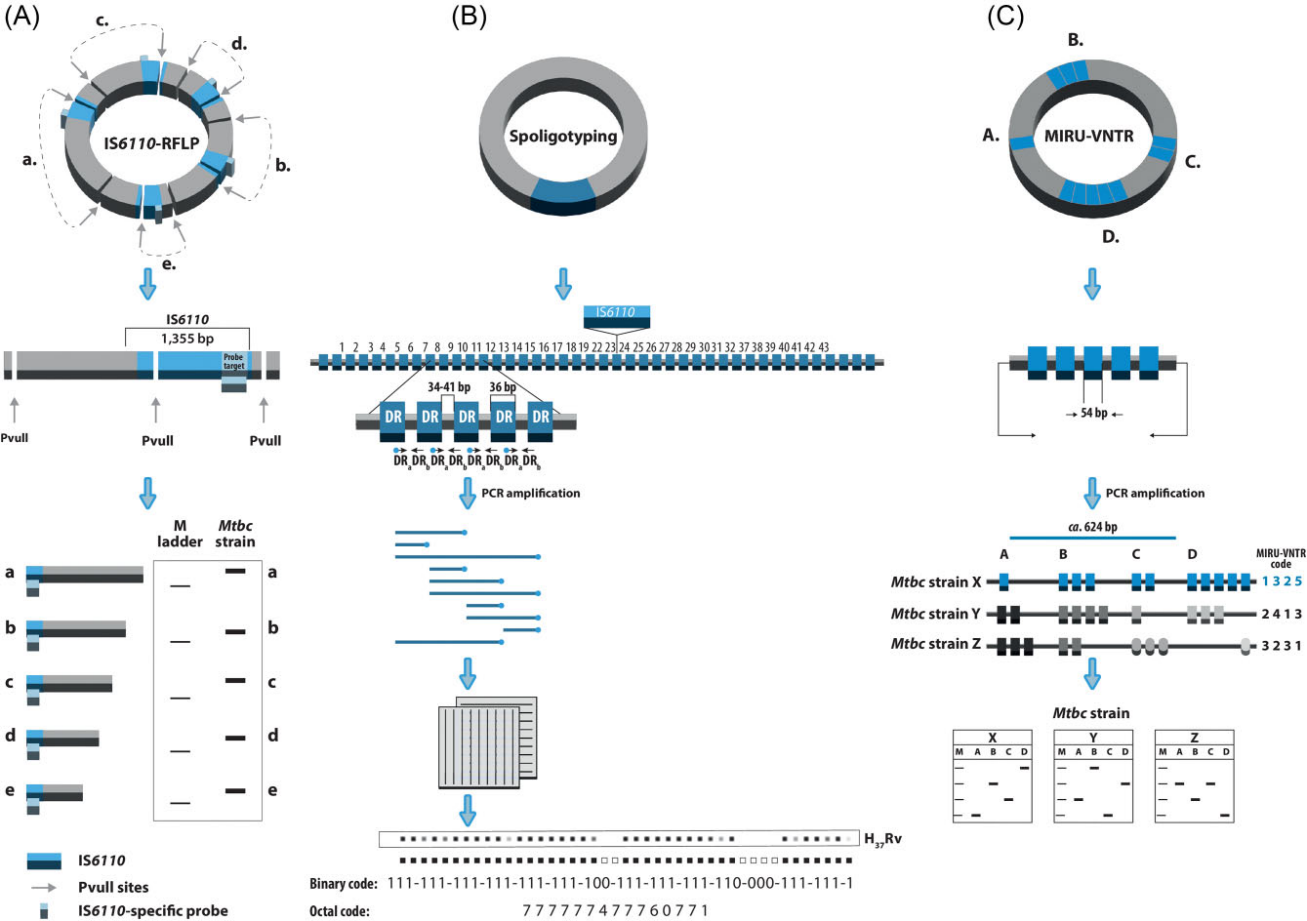
Schematic representation of three typing schemes used for *M. tuberculosis* strains. (A) Technically, the IS6110 genotyping is a Restriction Fragment Length Polymorphism (RFLP)-based method and involves genomic DNA digestion with PvuII endonuclease, electrophoretic separation of the fragments thus produced, and their hybridization with a peroxidase-labeled probe complementary to the 3′ end of the IS6110, allowing each copy of the sequence to be visualized as a separate band on an autoradiogram. (B) The standard spoligotyping procedure begins with PCR amplification of the entire DR region using two inversely oriented primers, complementary to short DR sequences, with one primer being biotinylated to make all PCR products labeled. These products are then hybridized to a membrane with a set of 43 immobilized, covalently bound synthetic oligonucleotides, corresponding to unique spacer sequences identified in either *M. tuberculosis* H37Rv or *M. bovis* BCG strains. Afterward, the membrane is incubated with a streptavidin-peroxidase or streptavidin-alkalic phosphatase conjugate, and the hybridization signals are detected by chemiluminescence. Strain-specific patterns (spoligotypes) are visualized autoradiographically by exposing the membrane to X-ray film. The presence or absence of a given spacer is represented by black squares (“1”) or blank spaces (“0”), respectively. Thus, a spoligotype is expressed as a 43-digit binary code, which can further be converted into octal code. (C) The MIRU-VNTR typing technique involves two major steps: PCR amplification of each MIRU-VNTR locus, with primers complementary to their flanking regions and analysis of thus produced amplicons, resolved electrophoretically. The number of tandem repeat units, at each locus, is deduced from the amplicon size, in relation to the known size of the repeat unit within the specific locus. The final result is a multidigit numerical code (MIRU-VNTR code), corresponding to the repeat number at each locus.

The insertion sequence *6110* (IS*6110*) was among the first genetic elements used as a marker for strain typing of *M. tuberculosis* (Thierry et al. 1990). IS*6110* is a 1355 bp-long IS3 family sequence, uniquely found in MTBC. It usually occurs in multiple copies ranging from 0 to 25 (five copies are presented), dispersed across the entire genome. The copy number and their chromosomal location determine the high level of marker’s polymorphism (Fig. 2A). The IS*6110*-RFLP was duly standardized, and several local or international databases were constructed (Van Embden et al. 1993, Heersma et al. 1998, Crawford et al. 2002), leading it to be considered as a gold standard for *M. tuberculosis* genotyping, as of the early 2000s (Kremer et al. 1999, Clark et al. 2006, Bifani et al. 2009). The IS*6110*-RFLP technique is highly discriminatory and generates profiles that are stable over time, yet whose rate of change allows detection of ongoing transmission events (Fang et al. 1998, Yeh et al. 1998, de Boer et al. 1999, Niemann et al. 2000). Apart from the identification of TB transmission chains and investigation of TB outbreaks (Valway et al. 1998, Kubín et al. 1999, Diel et al. 2004, Ruddy et al. 2004, Devaux et al. 2009), it was IS*6110*-RFLP that allowed, for the first time, to differentiate relapse from reinfection (Van Embden et al. 1993, van Rie et al. 1999) or trace the source of laboratory cross-contamination (Van Duin et al. 1998).

Despite its utility, the IS*6110*-RFLP typing system has several limitations, including its labor-intensive procedure, culture dependency, requirement for high DNA yield (>1 µg), need for advanced computer software, skilled personnel, and the lack of inter-laboratory reproducibility due to variations in assay conditions and interpretation of banding patterns (Van Soolingen and Arbeit 2001, Braden et al. 2002). Additionally, it has low discriminatory power for isolates with five or fewer IS*6110* copies, common in certain Asian regions where such isolates make up 47%–72% of circulating TB bacilli (Das et al. 2005, Rienthong et al. 2005, Joseph et al. 2013). These drawbacks led to a preference for Polymerase Chain Reaction (PCR)-based techniques, which often targeted IS*6110* (Friedman et al. 1995, Otal et al. 1997, Reisig et al. 2005, Thorne et al. 2007), but none of these methods achieved widespread adoption due to the lack of standardized performance measures and reference databases for cross-laboratory comparisons. Recent improvements in IS*6110*-based typing, such as the IS*6110*-5′3′FP (for IS6110 5′ and 3′ fluorescent polymorphisms) and semiautomated IS*6110*-*Pvu*II systems (tailored to the RiboPrinter microbial characterization system, DuPont Molecular Diagnostics, USA), offer better discriminatory power and technical flexibility, as well as improved throughput, reproducibility, and data portability (Thabet et al. 2014, Said et al. 2016, Dekhil et al. 2018). Nonetheless, high setup and maintenance costs may still deter most potential users.

To sum up, despite its high discriminatory power, limitations such as labor-intensive protocols and low reproducibility have led to decline of IS*6110*-RFLP in favor of PCR-based methods. Yet, regardless of the rise of WGS as the current gold standard for *M. tuberculosis* genotyping, IS*6110*-RFLP continues to be used for local TB investigations, either alone (Diaz et al. 2001, Razanamparany et al. 2002, Chauhan et al. 2007, Pescarini et al. 2018, Essahale et al. 2024), or in combination with other methods, like spoligotyping and MIRU-VNTR typing, particularly in low-income countries (Groenheit et al. 2011, Peres et al. 2018, Ei et al. 2019, Pokam et al. 2019, Chisompola et al. 2021).

### Spoligotyping

Spacer oligonucleotide typing, or spoligotyping (illustrated in Fig. 2B), is a widely used method for *M. tuberculosis* genotyping. It targets polymorphisms in the Clustered Regularly Interspersed Short Palindromic Repeats (CRISPR) in the Direct Repeat (DR) locus, consisting of 36-bp repeats interspersed with non-repetitive, 35–41-bp spacers, whose variability provides discriminatory power (Hermans et al. 1991, Groenen et al. 1993). Spoligotyping detects the presence or absence of 43 spacers selected from the *M. tuberculosis* H37Rv (spacers 1–19, 22–32, and 37–43) and *M. bovis* BCG vaccine strain P3 (spacers 20–21 and 33–36), yielding binary results suited for database portability and inter-laboratory comparisons (Groenen et al. 1993, Kamerbeek et al. 1997), as well as identification of members of the MTBC at both species and subspecies levels (Plikaytis et al. 1993, Kremer et al. 2004). Major databases include SpolDB4 (Brudey et al. 2006), SITVITWEB (Demay et al. 2012), and its 2019 update (Couvin et al. 2019), along with online tools like SPOTCLUST (Vitol et al. 2006), SpolLineages, and SpolSimilaritySearch (Couvin et al. 2017, 2020). These enable global tracking of TB genotypes (Eldholm et al. 2006, Ani et al. 2010, Dong et al. 2010, Tilahun et al. 2018).

Spoligotyping is fast, cost-effective, and highly sensitive, requiring only 10 fg of DNA, equivalent to the quantity from 2 to 3 bacterial cells (Jagielski et al. 2016). As a culture-independent method, it can be performed on diverse sample types, including TB-positive smears, paraffin-embedded tissue sections, or paleopathological specimens (Van Der Zanden et al. 1998, Zink et al. 2003, Schewe et al. 2005, Molina-Moya et al. 2018). Though initially used alone (De La Salmonière et al. 1997, Heyderman et al. 1998, Niang et al. 1999, Soini et al. 2000, Mistry et al. 2002, Puustinen et al. 2003, Augustynowicz-Kopeć et al. 2008), spoligotyping was unable to accurately assess the epidemiological links between TB cases (De La Salmonière et al. 1997, Goyal et al. 1997, Cronin et al. 2001). It is therefore combined with higher-resolution methods like IS*6110*-RFLP or MIRU-VNTR typing for enhanced epidemiological insights (Diaz et al. 1998, Cowan et al. 2005, Clark et al. 2006, Joseph et al. 2013, Bouklata et al. 2015, Jagielski et al. 2015, Ribeiro et al. 2015, Bakuła et al. 2019). Nevertheless, due to financial and/or organizational reasons, some laboratories, especially in developing countries, still rely on spoligotyping only (Zewdie et al. 2016, Elegail et al. 2018, Ramazanzadeh et al. 2020, Bellad et al. 2022, Hussien et al. 2022). Attempts to improve its discriminatory power, such as second-generation spacers (Van Embden et al. 2000, Van der Zanden et al. 2002), did not significantly increase the discriminatory resolution for *M. tuberculosis* (Van der Zanden et al. 2002, Kremer et al. 2005).

Efforts to improve technical aspects of spoligotyping involved (i) attempts to increase its high-throughput capacity, (ii) mitigate interpretative ambiguities with manual reading of the membrane, and (iii) expedite the turnaround time for obtaining results. Advanced detection techniques, such as Luminex technology, where the spacer probes are immobilized on microspheres and detected, upon hybridization, with PCR products and fluorochrome-mediated binding, by laser-based flow cytometry (Cowan et al. 2004, Zhang et al. 2010), were attempted. Additional developments included (i) advanced Luminex analyzer (MAGPIX) based on the use of magnetic beads and light-emitting-diode/charge-coupled-device image-based detection system (Ocheretina et al. 2013); (ii) Matrix Assisted Laser Desorption Ionization - Time of Flight Mass Spectrometry (MALDI-TOF MS) for spoligotype detection, with the hybridization step replaced with a multiplexed primer extension assay (Honisch et al. 2010); and (iii) variety of microarray platforms designed for spoligotyping to optimize its performance and efficiency (Song et al. 2007, Gomgnimbou et al. 2012, Bespyatykh et al. 2014). Though promising with respect to working time and data processing, these assays remain limited by high costs (Honisch et al. 2010, Ocheretina et al. 2013, Bespyatykh et al. 2014), especially for middle- and low-income countries. Newer innovations, such as a new, three-reaction, one-step real-time PCR-based McSpoligotyping and its refined, single-tube version (MeltArray-based spoligotyping), proposed as a rapid and reliable alternative for the conventional spoligotyping protocol, might have the potential to be implemented in resource-limited settings (Zeng et al. 2018, Xia et al. 2024).

*In silico* spoligotyping tools such as SpoTyping (Xia et al. 2016), SpolPred (Coll et al. 2012), SpolPred2 (Napier et al. 2023), lorikeet (Cohen et al. 2015), and TGS-TB (Sekizuka et al. 2015) offer simplified workflows and compatibility with WGS. Note that the latter provides *in silico* genotyping for spoligotyping as well as other typing formats, including the analysis of IS*6110* insertion sites and customized VNTR loci. *In silico* spoligotyping has been employed in several WGS-based studies, allowing backward compatibility of WGS with molecular spoligotyping (Coll et al. 2012, Hijikata et al. 2017, Gautam et al. 2018, Bogaerts et al. 2021, Genestet et al. 2022, Bakuła et al. 2023a, Napier et al. 2023).

Direct comparisons between conventional and *in silico* spoligotyping are discouraged due to factors like sequence read quality and bioinformatic criteria. *In silico* methods may miss changes in the DR locus, such as IS*6110* insertions, but improve accuracy in depicting strain relatedness (Bakuła et al. 2023a).

Despite limitations, including homoplasy and being less discriminatory for closely related strains (Reyes and Tanaka 2010, Reyes et al. 2012), spoligotyping remains valuable for assessing genetic diversity and phylogenetic relationships among *M. tuberculosis* strains (Liang et al. 2020, Bakuła et al. 2023b, Yin et al. 2023). Even after three decades, spoligotyping remains a largely used method in the investigation of genetic diversity and transmission dynamics of TB bacilli circulating within specific populations and settings (Razo et al. 2018, Shi et al. 2018, Ramazanzadeh et al. 2020, Hussien et al. 2022, Yin et al. 2023, Rudeeaneksin et al. 2024, Valencia-Trujillo et al. 2024). With nearly 1800 PubMed articles referencing it as of December 2024, it continues to feature prominently in TB molecular epidemiology.

### MIRU-VNTR genotyping

Minisatellite-like VNTR loci were identified in *M. tuberculosis* genomes in the late 1990s (Supply et al. 1997, Frothingham and Meeker-O'Connell 1998). These 40–100-bp mycobacterial interspersed repetitive units (MIRUs) are scattered across 41 chromosomal locations (Supply et al. 2000). A 12-locus MIRU-VNTR typing scheme (shown in Fig. 2C) was developed for genotyping (Mazars et al. 2001), offering high-throughput analysis through PCR and gel or capillary electrophoresis (Supply et al. 2001, Nikolayevskyy et al. 2016b, Tafaj et al. 2020). Further, its digitized results allowed easy global database integration, aiding researchers. Remarkably, the 12-locus MIRU-VNTR typing is more discriminatory than spoligotyping and IS*6110*-RFLP for IS*6110* low-copy strains (Mazars et al. 2001, Cowan et al. 2002, Lee et al. 2002); however, it is less effective for high-copy strains unless combined with another typing method (Blackwood et al. 2004, Cowan et al. 2005, Gopaul et al. 2006).

To enhance resolution, a standardized 24-locus format, including a subset of 15 discriminatory loci, was proposed, suitable for epidemiology and phylogenetic studies (Supply et al. 2006, Oelemann et al. 2007, Allix-Béguec et al. 2008). However, homoplasy issues necessitate lineage-specific locus sets (Comas et al. 2009, Maghradze et al. 2022), particularly for Beijing lineage strains necessitating hypervariable loci (Iwamoto et al. 2007, Mokrousov et al. 2008, Comas et al. 2009, Velji et al. 2009, Allix-Béguec et al. 2014), different from the standard 15- or 24-loci formats. Thus, a consensus set of 4 hypervariable loci was proposed as an adjunct to standard typing for Beijing clonal clusters (Allix-Béguec et al. 2014).

MIRU-VNTR typing has largely replaced IS*6110*-RFLP as the gold standard due to its technical advantages (Merker et al. 2017), and has been applied to studies on TB transmission (Van Deutekom et al. 2005, Oelemann et al. 2007, Maes et al. 2008, Bidovec-Stojkovic et al. 2011, Mansoori et al. 2018, Chen et al. 2022, Maghradze et al. 2022), discriminate relapses from reinfections (Afshar et al. 2019, Maghradze et al. 2019, Shao et al. 2021), identify mixed infections (Wang et al. 2015, Kargarpour Kamakoli et al. 2020, Micheni et al. 2022), and laboratory cross-contaminations (Martín et al. 2008).

Used alongside spoligotyping, it aids in unraveling genetic diversity and evolutionary relationships (Sola et al. 2003, Chaoui et al. 2014, Bouklata et al. 2015, Shi et al. 2018), leveraging databases like MIRU-VNTRplus that allow phylogenetic comparisons between worldwide samples of TB bacilli populations (Allix-Béguec et al. 2008, Weniger et al. 2010). Despite advancements in sequencing, MIRU-VNTR remains an efficient tool for TB epidemiology, with emerging *in silico* approaches promising to replace conventional methods (Rajwani et al. 2018, Maeda et al. 2020). Digital MIRU-VNTR typing, performed on complete or draft genome sequences, is expected to eventually replace the conventional procedure in future, similar to spoligotyping.

### Whole-genome sequencing

The advent of WGS has transformed the study of pathogen genetics, including tubercle bacilli, delivering significant advancements in TB epidemiology over the past two decades (Box 1). WGS surpasses spoligotyping, MIRU-VNTR, and other methods in determining genetic relatedness among *M. tuberculosis* strains (Nikolayevskyy et al. 2019). Two key WGS approaches are widely used: single nucleotide polymorphism (SNP) variant calling, which identifies single nucleotide differences with a reference genome (e.g. *M. tuberculosis* H37Rv, GCF_000195955.2) and provides robust phylogenetic markers due to the rarity of SNP events and low homoplasy (Stucki and Gagneux 2013, Gagneux 2018), and gene-by-gene typing, which detects allelic variations in core or accessory genes, extending the multi-locus sequence typing (MLST; Maiden et al. 2013).

Box 1. The first generation of WGS is mostly represented by Sanger’s chain termination-based sequencing method (Sanger et al. 1977). This method uses dideoxynucleotides, which interrupt the elongation of DNA strands during replication, making it possible to produce reading sequences with a maximum length of a few hundred nucleotides. The ABI 370 was the first commercially available automated sequencer by Applied Biosystems Co. It used fluorescently labeled dideoxynucleotides and capillary electrophoresis to perform the sequencing automatically, as designed by Sanger.

Soon after the first application of WGS for MTB with the Sanger method (Cole et al. 1998), a need for faster and more cost-effective alternatives emerged. This led to the development of next-generation sequencing (NGS) technologies, which have surpassed Sanger sequencing by enabling rapid and simultaneous sequencing of thousands or millions of DNA fragments. These technologies differ on various parameters, such as DNA extraction methods, library preparation (including fragment size), sequencing strategy, and base-pair detection system. Based on these variables, NGS techniques are classified as second- and third-generation (Tyler et al. 2016).

**Figure 3.**
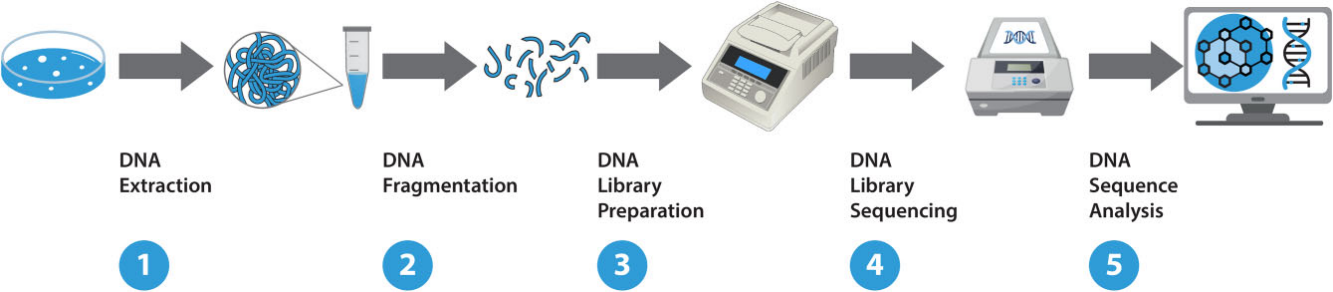
Key steps of the whole-genome sequencing process.

Unlike traditional Sanger approach, second-generation sequencing (SGS) methods have the ability to perform massive parallel sequencing of multiple DNA fragments (Tucker et al. 2009). Numerous SGS platforms have become available, including (i) Roche’s 454 sequencing method, where the sequence is determined by detecting pyrophosphate release upon nucleotide addition to the DNA template (Margulies et al. 2005), (ii) Ion Torrent sequencing by identifying hydrogen ion release during DNA synthesis (Parson et al. 2013), (iii) Illumina sequencing uses reversible dye terminators in a sequencing-by-synthesis method, with repeatedly added fluorescently labeled nucleotides to build up the reads, (iv) ABI SOLiD sequencing (sequencing by oligonucleotide ligation and detection), which employs a ligation-based approach with reversible terminators for DNA sequence determination. However, SGS methods have several drawbacks, such as the highly fragmented reads, which make reconstructing the genome difficult to perform, especially for genomes with a wide range of repeated regions, and with the GC-rich fragments poorly amplified and under-represented (Niedringhaus et al. 2011, Liu et al. 2012). Third-generation sequencing (TGS) technologies represent the latest advancements in DNA sequencing, overcoming the limitations of the previous generations. These technologies provide long-read sequencing capabilities, enabling the sequencing of much larger DNA fragments without the need of upstream PCR amplification. Among TGS platforms are PacBio sequencing, which uses a single-molecule, real-time approach with fluorescently labeled nucleotides, enabling long-read sequencing of DNA fragments of up to tens of kb in length (Rhoads and Au 2015) or Oxford Nanopore sequencing, based on nanopore technology, where a single-stranded DNA molecule passes through a nanopore, and changes in electrical current are measured to determine the DNA sequence (Lu et al. 2016). This technology is currently the most advanced in the field of sequencing and genotyping of MTBC. All WGS approaches follow the same general path depicted in Fig. 3.

Early studies demonstrated WGS’s superiority over IS*6110*-RFLP and MIRU-VNTR for inferring epidemiological links and genetic relatedness among *M. tuberculosis* isolates (Schürch et al. 2010, Gardy et al. 2011). A systematic review confirmed WGS’s higher discriminatory power compared to classical genotyping (Nikolayevskyy et al. 2016a). WGS frequently subdivides MIRU-VNTR clusters, ruling out false transmission events. MIRU-VNTR clustering rates were overestimated by 7%–92%, particularly for monomorphic lineages like the Beijing family (Gurjav et al. 2016, Stucki et al. 2016, Meehan et al. 2018, Wyllie et al. 2018, Alaridah et al. 2019). Additionally, WGS identified transmission events missed by conventional epidemiological methods (Nikolayevskyy et al. 2016a, 2019).

The SNP threshold for defining transmission clusters is critical. A 5-SNP cut-off, based on within-strain divergence over three years, is often used to indicate recent transmission, while >12 SNPs suggest no direct link (Walker et al. 2013). Subsequent studies validated the 5-SNP threshold for epidemiologically linked cases (Casali et al. 2016, Norheim et al. 2017, Iwamoto et al. 2023; Zhang et al. 2023). However, appropriate SNP thresholds vary depending on factors like strain diversity, read quality, within-host diversity, and amplification steps (Hatherell et al. 2016). Some studies identified links at 2–3 SNPs (Roetzer et al. 2013, Walker et al. 2014, 2018), while others found connections even at >12 SNPs, which is defined as the upper limit of genomic relatedness between epidemiologically related individuals (Luo et al. 2014, Nikolayevskyy et al. 2016a, Jajou et al. 2018, Cancino-Muñoz et al. 2022, Xiao et al. 2024).

WGS and SNP differences are used to distinguish relapse from reinfection, with SNP distances varying significantly between original and new infections (0–8 vs. >1000 SNPs) (Bryant et al. 2013, Witney et al. 2017). SNP-based WGS protocols face challenges such as sequencing errors, base-calling inaccuracies, and incomplete genome assembly due to repetitive elements (Meacham et al. 2011, Ahmad et al. 2021). Additionally, the lack of global data standardization and diverse analysis pipelines hinder inter-laboratory comparisons (Merker et al. 2015, Kohl et al. 2018a, Meehan et al. 2019). To address these limitations, core genome multilocus sequence typing (cgMLST) offers a standardized approach, using uniform allele numbering to describe genetic variation based on a defined scheme of loci and alleles (Maiden et al. 2013, Kohl et al. 2014, 2018a).

The initial cgMLST scheme included 3257 loci shared among reference genomes of Lineages 4 and 6 of *M. tuberculosis*, and *M. bovis*, covering 80% of the coding capacity of *M. tuberculosis* H37Rv (Kohl et al. 2014, Merker et al. 2017, Kohl et al. 2018a). A refined scheme with 2891 core genes was later developed using the cgMLST definer tool of “SeqSphere+” software (Kohl et al. 2018a) with a broader set of genomes, including all MTBC lineages and isolates from animal-adapted species, such as *M. bovis, M. caprae, M. microti*, and *M. pinnipedii*, achieving 97.4% coverage compared to 94.2% in the initial scheme (Kohl et al. 2018a, O'Toole 2018). This updated scheme has been widely applied in several studies (Peker et al. 2021, Leong et al. 2022, Mekonnen et al. 2023, Quan et al. 2024; Song et al. 2024). In cgMLST, each locus is assigned a unique allele number, forming a sequence type (ST) for strain identification (Maiden et al. 2013, Jajou et al. 2019a). A threshold of more than 12 allele differences is recommended to exclude recent transmission, with allele change rates within the 2891 loci set, being comparable to SNPs (∼0.5 changes/year) (Gagneux 2017, Kohl et al. 2018a). While reliable, cgMLST showed slightly lower discriminatory power in regions with low genetic diversity (Peker et al. 2021). Software tools like RIdom SeqSphere (Jünemann et al. 2013) and databases like TB Portals and GenTB facilitate standardized data analysis, enhancing TB outbreak investigations and epidemiological studies (Rosenthal et al. 2017, Gröschel et al. 2021). This makes cgMLST a powerful tool for understanding TB epidemiology, including outbreak investigations, disease control, and assessing risk factors (Maiden et al. 2013, Kohl et al. 2014, Satta et al. 2017, Jajou et al. 2019a, Jones et al. 2019, Merker et al. 2021, Mudliar et al. 2022).

WGS technology has significantly advanced the diagnosis and monitoring of drug-resistant TB, offering rapid and accurate detection of resistance-associated mutations (Papaventsis et al. 2017, Veziris et al. 2017, Walker et al. 2017, Acharya et al. 2020, Ramirez et al. 2020). Compared to traditional phenotypic tests, WGS demonstrates high sensitivity in predicting drug resistance, particularly for first-line anti-TB drugs, often exceeding 90% accuracy (Shea et al. 2017, The CRyPTIC Consortium and the 100 000 Genomes Project 2018, Jajou et al. 2019b, Wu et al. 2020). Under continuous selective antibiotic pressure on TB bacilli, the mutation rate can increase significantly, from 0.5 up to 4.3 SNPs per genome per year (Walker et al. 2013). WGS has been crucial in tracking resistance evolution (Eldholm et al. 2014, Manson et al. 2017, Jajou et al. 2019b, Li et al. 2022), predicting treatment outcomes (He et al. 2020, Katale et al. 2020), and deciphering resistance mechanisms, including for newer drugs like bedaquiline and Delamanid (Ramirez et al. 2020, Chesov et al. 2022). Supported by an ever-expanding battery of bioinformatics tools (e.g. TBprofiler, Mykrobe Predictor, CASTB, and Resistance Sniffer), WGS is a suitable method for investigating TB drug resistance (Iwai et al. 2015, Hunt et al. 2019, Phelan et al. 2019, Muzondiwa et al. 2020, Lam et al. 2021).

WGS has also markedly improved the resolution of *M. tuberculosis* strain genotyping, enhancing the ability to detect transmission clusters accurately (Meehan et al. 2019). While its wider routine use is still limited by cost, turnaround time, and required expertise, WGS is expected to become the new gold standard for studying TB transmissions and surveillance. Ongoing technological advancements aim to increase throughput capacity while reducing complexity, potentially making WGS more accessible to smaller laboratories. However, global implementation may take longer for TB due to its concentration in resource-limited settings.

## Chapter 2: an overview of software tools and databases for analyzing TB molecular data, and examples of their use

Molecular typing techniques are essential for developing TB-specific software tools and databases, as they provide detailed genetic insights into M. tuberculosis strains, including transmission routes, evolutionary patterns, and drug resistance mechanisms. These data support monitoring and surveillance efforts, requiring regular updates to software and databases to keep pace with advances in WGS methods. This section briefly highlights current tools and databases used to elucidate TB molecular epidemiology, with examples demonstrating their applications.

### Software tools for analyzing TB WGS data

Various software tools and bioinformatics workflows have been developed to analyze TB molecular and WGS data, including raw sequencing reads and assembled genomes. Variant calling, a key method for SNP-based comparative genomics, helps differentiate isolates using reference genomes. These workflows aim to elucidate TB transmission, drug resistance mechanisms, and lineage prediction. Genomic data also enable broader analyses. A recent preliminary list of TB-specific tools includes MTBseq, PhyResSE, SAM-TB, TB-Profiler, TransFlow, and Mykrobe predictor TB (Couvin et al. 2021).

- MTBseq is an automated pipeline for mapping, variant calling, and detecting drug resistance determinants, enabling detailed phylogenetic classification of MTBC isolates from Illumina WGS data (Kohl et al. 2018b).
- PhyResSE is a web tool that identifies *M. tuberculosis* lineage and drug resistance from WGS data, integrating tools like FastQC, BWA, QualiMap, SAMtools, and others for quality checks before reporting lineage and resistance patterns (Feuerriegel et al. 2015).
- SAM-TB predicts MTBC drug resistance, identifies species, and assesses inter-strain genetic relatedness, including mixed samples with NTM and MTBC. It offers a user-friendly online platform (Yang et al. 2022).
- TB-Profiler aligns reads to the H37Rv genome using bowtie2, BWA, or minimap2, calls variants using bcftools, and compares them to a drug-resistance database (tbdb). It supports lineage detection, spoligotyping, SNP distance computation, and more (Phelan et al. 2019).
- TransFlow is a modular TB transmission analysis workflow that processes raw sequencing data to infer transmission clusters, networks, and risk factors, generating summary reports with visualization (Pan et al. 2023).
- Mykrobe predictor TB rapidly analyzes bacterial WGS data to predict drug resistance, requiring no expertise and operating offline on standard devices. It has been extensively validated on thousands of samples (Hunt et al. 2019).

These tools offer fast and accurate WGS-based TB analysis, as evaluated by regular assessment of performance (Morey-León et al. 2023). With the advent of artificial intelligence (AI), new software tools, such as GenTB, tend to integrate AI approaches into their algorithms to enhance prediction accuracy (Gröschel et al. 2021). As summarized in Table 1, WGS-based software tools such as *in silico* platforms for spoligotyping and/or MIRU-VNTR typing are now designed to replace classical typing methods (Morey-León et al. 2023). The selection criteria for these tools were accessibility and the ability to predict spoligotyping and/or MIRU-VNTR patterns.

**Table 1.** Non-exhaustive list of software tools used for *in silico* spoligotyping and/or MIRU-VNTR typing from WGS TB.

Software tool name	Short description	Link or reference
SpolPred	Software tool used for prediction of spoligotypes from short genomic sequences	(Coll et al. 2012)
SpolPred2	An updated version of SpolPred that has been integrated into TB-Profiler	https://github.com/GaryNapier/spolpred; https://github.com/jodyphelan/TBProfiler (Napier et al. 2023)
SpoTyping	Fast and accurate *in silico Mycobacterium tuberculosis* spoligotyping from sequence reads	https://github.com/xiaeryu/SpoTyping-v2.0 (Xia et al. 2016)
Miru-Hero	Mycobacterial interspersed repetitive unit heuristics for evaluation of repeats and their ordinal	https://gitlab.com/LPCDRP/miru-hero
Galru	Long read spoligotyping for *Mycobacterium tuberculosis*	https://github.com/quadram-institute-bioscience/galru (Page et al. 2020)
MIRUReader	In-silico MIRU-VNTR typing using long reads	https://github.com/phglab/MIRUReader (Tang and Ong 2020)
MIRU-profiler	Performing digital 24-loci MIRU-VNTR typing for Mycobacterium tuberculosis	https://github.com/rahimrajwani/MIRU-profiler (Rajwani et al. 2018)
lorikeet	Digital spoligotyping of MTB strains from Illumina read data	https://github.com/AbeelLab/lorikeet (Cohen et al. 2015)
CRISPRbuilder-TB	CRISPR reconstruction based directly on short read sequences in *M. tuberculosis*	https://github.com/cguyeux/CRISPRbuilder-TB (Guyeux et al. 2021)

In conclusion, software tools are invaluable for analyzing WGS data to identify and detect specific patterns. These tools can facilitate the creation of dedicated databases by processing output/result files or utilizing internal scripts. Additionally, specialized programs can be developed to establish automated routines that regularly update databases based on predefined criteria or rules.

### Databases for analyzing TB WGS data

Databases are essential for efficiently managing and sharing large-scale data in scientific fields, including TB research. Over the past two decades, numerous TB databases have been developed, offering multi-level information on strains from global, national, or regional studies. Key databases for classical TB genotyping include MIRU-VNTRplus and SpolDB/SITVIT, the latter maintained by the Institut Pasteur de la Guadeloupe. Data entry is automated through scripts, but manual curation ensures accuracy. These databases integrate diverse data (e.g. patient demographics, drug resistance, phylogeographic, epidemiologic, genetic, and available socio-demographic data), enabling large-scale comparisons and benchmarking. Table 2 highlights examples of databases available for TB data analysis, based on their accessibility and their relationship with MTBC data. These databases are valuable for resolving TB phylogeographic diversity at local and global levels, integrating epidemiological and demographic data. Geo-mapping combined with phylogeographic analysis enables effective monitoring of clonal transmission patterns, enhancing TB surveillance and control efforts.

**Table 2.** Non-exhaustive list of commonly used TB databases.

Databases	Description	Reference/Link
CPLP-TB	Database aiming to to facilitate exchange of molecular epidemiological data and thus enable the tracking of important MTB clones across the Lusophone space	http://cplp-tb.ff.ulisboa.pt./ (Perdigão et al. 2019)
GMTV	Database integrating clinical, epidemiological and microbiological description with genome variations based on WGS data	(Chernyaeva et al. 2014)
Mbovis.org	Database containing *M. bovis* Spoligotyping data	https://www.mbovis.org/ (Smith and Upton 2012)
Mycobrowser	Comprehensive genomic and proteomic data repository for pathogenic mycobacteria	https://mycobrowser.epfl.ch/ (Kapopoulou et al. 2011)
MycoDB.es	Spanish Database of Animal Mycobacterosis	http://www.vigilanciasanitaria.es/mycodb/ (Rodriguez-Campos et al. 2012)
MIRU-VNTRplus	Web tool for polyphasic genotyping of MTBC bacteria	http://www.miru-vntrplus.org/ (Weniger et al. 2010)
ReSeqTB	Collaborative effort for a centralized worldwide TB relational sequencing data platform	https://www.reseqtb.org/ (Starks et al. 2015)
SITVIT2	the sixth international multimarker database for studying MTBC genetic diversity and molecular epidemiology	http://www.pasteur-guadeloupe.fr:8081/SITVIT2/ (Couvin et al. 2019)
SITVITBovis	Database and mapping tool to get an improved overview of animal and human cases caused by *Mycobacterium bovis*	http://www.pasteur-guadeloupe.fr:8081/SITVIT_Bovis/ (Couvin et al. 2022)
TB-Annotator	Pipeline and database used for the reconstruction of a global TB history	(Senelle et al. 2023)
TBDB	Repository containing the scripts and data to generate all files required to run TBProfiler	https://github.com/jodyphelan/tbdb (Phelan et al. 2019)
tbvar	*Mycobacterium tuberculosis* variome resource	(Joshi et al. 2014)
TB Portals	Web-based platform for global drug-resistant-tuberculosis data sharing and analysis	https://tbportals.niaid.nih.gov/ (Rosenthal et al. 2017)

### Mapping global circulation, transmission patterns, and TB surveillance

**Figure 4.**
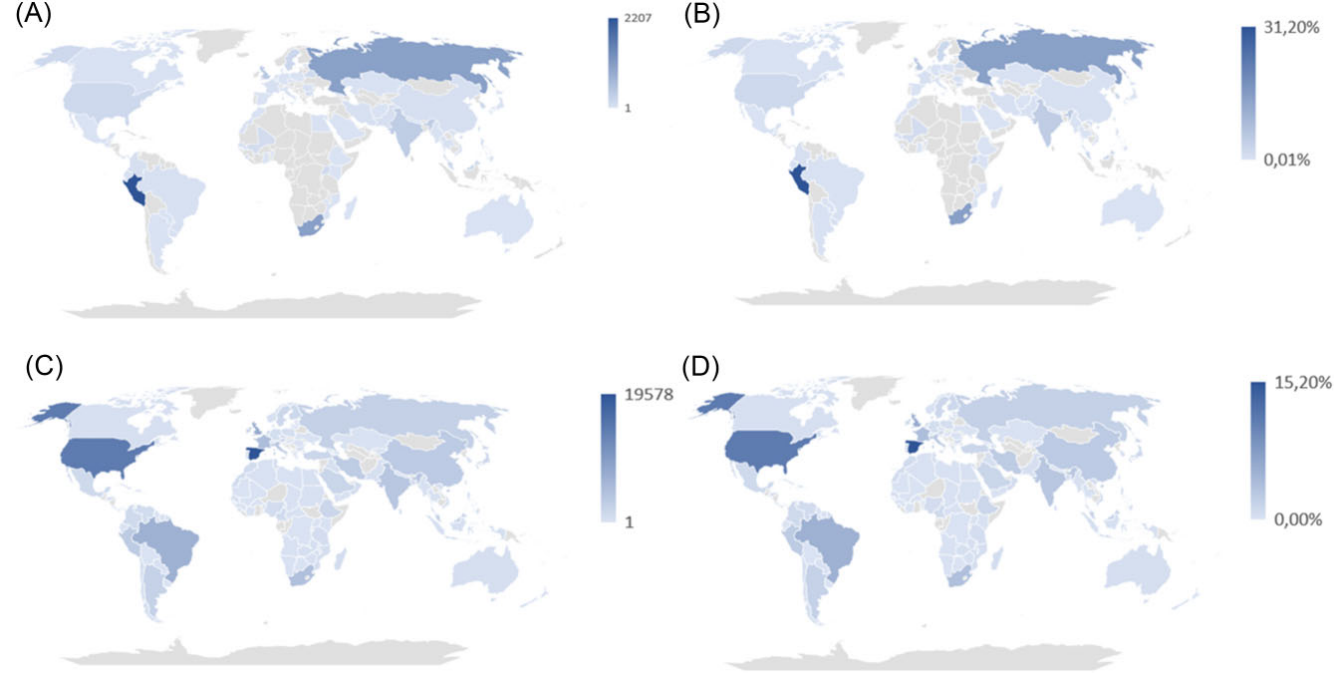
Intensity maps showing the distribution of RefSeq genome assemblies in terms of number (A) and percentage by country (B) contained in RefSeq repository (data collected in November 2023); and intensity maps showing the distribution of isolates contained in SITVITEXTEND database, in terms of number (C) and percentage by country (D).

Global mapping data provide valuable insights for TB surveillance and analysis across diverse contexts. Figure 4 illustrates the geographical distribution of TB strains based on NCBI’s RefSeq repository (Haft et al. 2024) and the SITVITEXTEND database. As of November 2023, RefSeq included 7057 genome assemblies, with distributions shown by isolate counts and percentages per country (Fig. 4A, B). Similar data from SITVITEXTEND (Fig. 4C, D) revealed disparities in genome availability, particularly in resource-limited regions such as Africa, the Caribbean, and Southeast Asia, where WGS adoption is limited. Note that the SITVIT database, with 128 000 isolates collected over 20 years through extensive collaborations, contains a significantly larger dataset.

**Figure 5.**
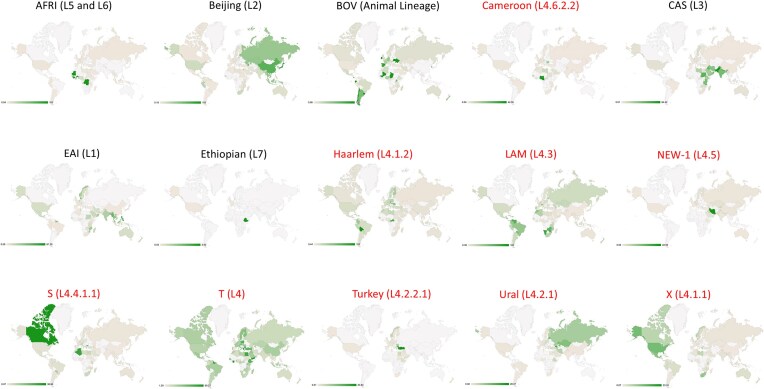
Distribution of main TB families (associated with SNP-based lineages/sublineages) contained in SITVITEXTEND. Families and lineages written in red represent Euro-American lineage isolates (i.e. Cameroon, Haarlem, LAM, NEW-1, S, T, Turkey, Ural and X).

Databases have enabled numerous studies on the distribution of TB phylogenetic lineages and families by country, region, or continent (Fig. 5). These studies highlight the geographical specificity of TB strains, with spoligotyping families in the SITVIT databases often used alongside SNP-based lineages to assess phylogeographic patterns. Table 3 shows the correspondence between spoligotyping families and SNP-based lineages (SNP barcode nomenclature). Notably, recently discovered lineages (Lineage 8, Lineage 9, and Lineage 10), clearly appear to be restricted to Africa (Ngabonziza et al. 2020, Coscolla et al. 2021, Guyeux et al. 2024).

**Table 3.** Correspondence between TB SNP-based lineages and spoligotyping families.

SNP-based lineage	Spoligotyping families
Lineage 1 (Indo-Oceanic)	East-African-Indian (EAI)
Lineage 2 (East-Asian)	Beijing
Lineage 3 (East-African-Indian)	Central Asian (CAS)
Lineage 4 (Euro-American)	Cameroon, Haarlem (H), Latin-American-Mediterranean (LAM), NEW-1, S, T, Turkey, Ural, and X
Lineage 5 (West-Africa 1)	AFRI 2 and AFRI 3
Lineage 6 (West-Africa 2)	AFRI 1
Lineage 7 (Ethiopian)	Ethiopian
Lineage 8 (African Great Lakes)	Not Defined
Lineage 9 (East Africa)	Not Defined
Lineage 10 (Central Africa)	Not Defined

**Figure 6.**
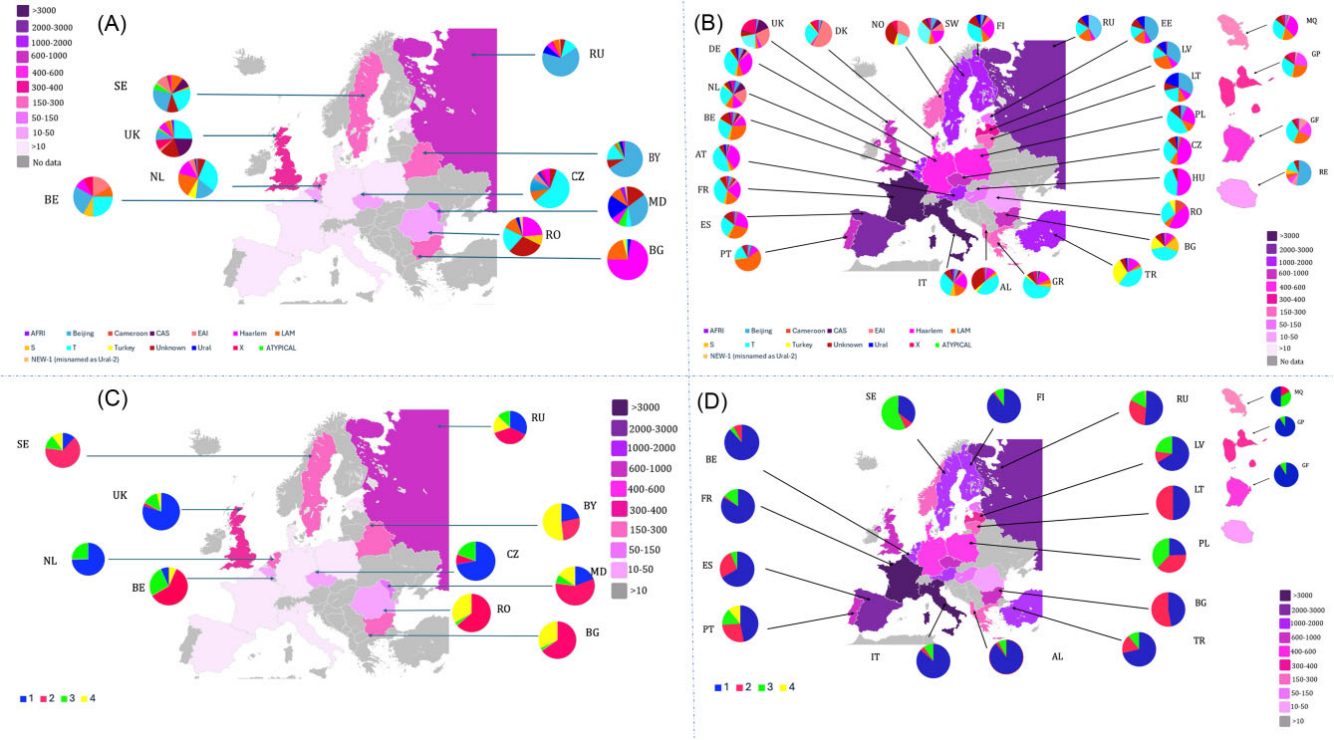
Maps showing TB families distribution in Europe for RefSeq repository (A) and SITVITEXTEND database (B); and for drug resistance distribution in RefSeq and SITVITEXTEND, respectively (C and D). 1–4 code numbers used in these maps (C and D) represent drug resistance profiles used in SITVIT databases. Note that two-letter country codes were used to identify countries based on ISO 3166-1 standard (https://en.wikipedia.org/wiki/ISO_3166-1_alpha-2). Countries have been colored according to the number of isolates (the darker the color, the higher the number of isolates).

A phylogeographic study of 21 574 TB strains (excluding *M. bovis*) from SITVITEXTEND revealed disparities across European countries. These strains were isolated from year 1890 to 2021 from 23 countries (Albania AL, *n* = 237; Austria AT, *n* = 1575; Belgium BE, *n* = 1369; Bulgaria BG, *n* = 639; Czech Republic CZ, *n* = 637; Denmark DK, *n* = 550; Estonia EE, *n* = 119; Finland FI, *n* = 1427; France FR, *n* = 3509; Germany DE, *n* = 455; Greece GR, *n* = 170; Hungary HU, *n* = 65; Italy IT, *n* = 3191; Latvia LV, *n* = 363; Lithuania LT, *n* = 200; Netherlands NL, *n* = 1355; Norway NO, *n* = 89; Poland PL, *n* = 523; Portugal PT, *n* = 722; Romania RO, *n* = 14; Spain ES, *n* = 2056; Sweden SE, *n* = 1409; United Kingdom GB, *n* = 900). Genomic data from RefSeq and SITVITEXTEND were analyzed using simpiTB, Miru-Hero, SpolLineages, and TB-Profiler to infer spoligotyping families and drug resistance profiles. Comparative analyses showed that four major spoligotyping families (T, Beijing, Haarlem, and LAM) were widespread, though proportions varied. Beijing lineage strains were predominant in eastern European countries, such as Russia, Estonia, Lithuania, Latvia, Belarus, and Moldova (Fig. 6A, B), and accounted for nearly 70% of strains in Russia and Belarus. Conversely, some countries in Northern and Western Europe, such as Sweden, the UK, the Netherlands, and Belgium, showed a distinct and heterogeneous distribution of *M. tuberculosis* families. Nevertheless, the proportion of the T family remains relatively constant in these countries, accounting for around 25% of MTBC isolates. In French overseas territories, the MTBC family distribution in Guadeloupe, Martinique, and French Guiana resembled Western Europe, while Reunion Island displayed a higher prevalence of Beijing strains (Fig. 6B).

Drug resistance was categorized using SITVITEXTEND codes:

Code 1: Pan-susceptible strains.
Code 2: MDR-TB (resistance to INH and RIF, ± other drugs).
Code 3: Resistance to other drugs.
Code 4: XDR-TB (MDR-TB + fluoroquinolone + any 1 of 3 injectable 2nd-line drugs (capreomycin, kanamycin, amikacin). Note that newer drugs have been introduced to treat TB disease for managing resistant strains, which are not shown in this study.

Note that a significant proportion of MDR-TB and XDR-TB strains was found mainly in the Eastern European countries (both in RefSeq and STIVITEXTEND databases), such as Russia, Latvia, Poland, Bulgaria, Moldova, and Romania. On the other hand, higher proportions of pansusceptible strains were observed in France, Italy, Albania, and Finland (Fig. 6C, D). In order to harmonize the data recorded, it would be recommendable to carry out studies on European countries for which we have little or no data such as Slovakia, Slovenia, and Switzerland.

**Figure 7.**
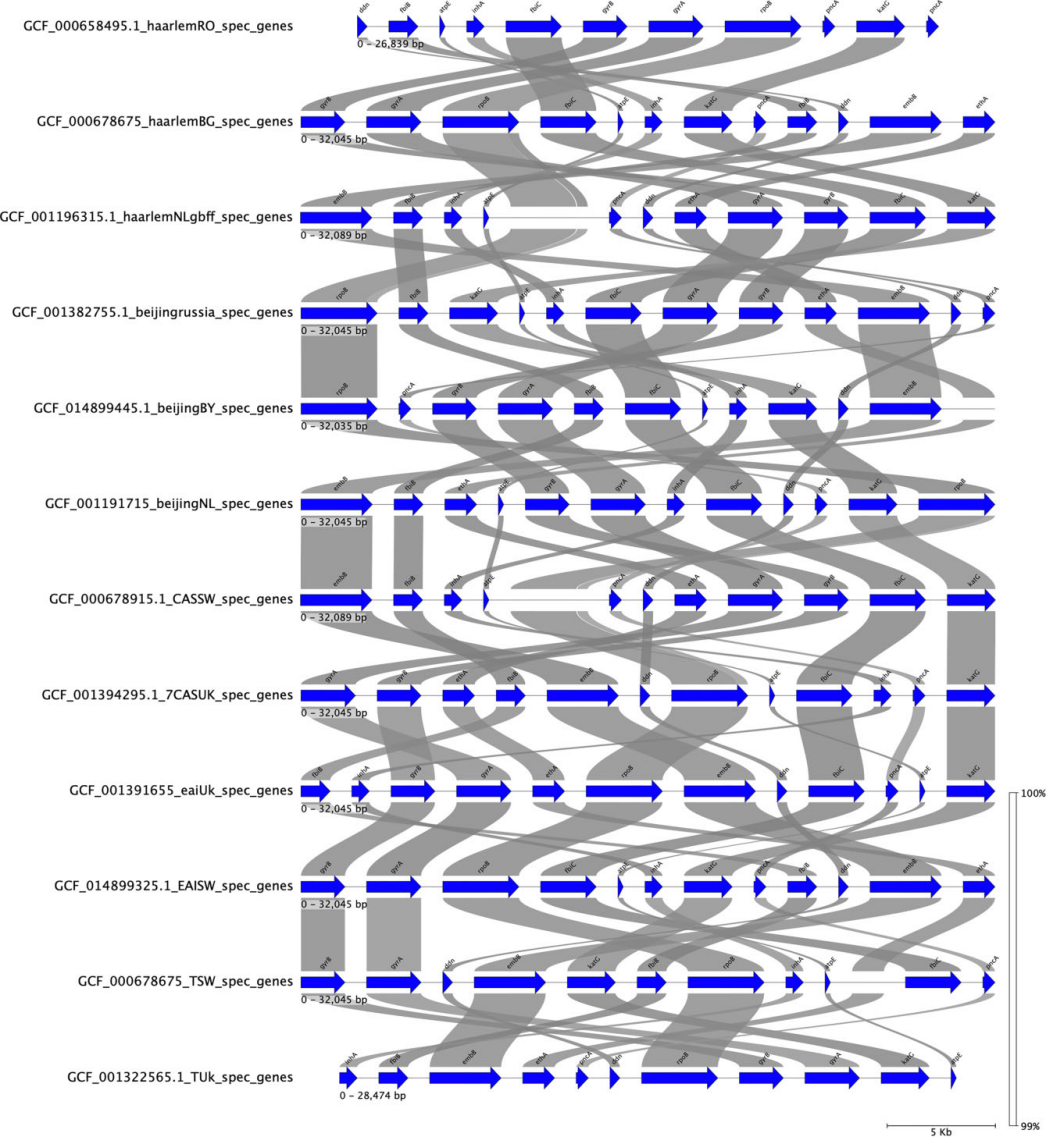
Synteny map showing the distribution and similarity between various drug resistance genes (e.g. rpoB, katG, ethA, inhA, pncA, embB, rrs, and gyrA) recovered from selected RefSeq genome assemblies.

Antibiotic resistance, particularly MDR and XDR TB, is a global public health issue affecting the management of TB. New strategies, including the use of specific software tools and databases, are essential for studying drug-resistant tuberculosis and improving data visualization. These tools help answer broader microbiological questions on TB transmission, drug resistance, virulence, and epidemiology. At the Institut Pasteur de la Guadeloupe, tools like getSequenceInfo and “getGenesFromGenBank.py” (https://github.com/karubiotools/getSequenceInfo) enable the extraction of specific genes from genome assemblies to study resistance gene acquisition across TB families (Moco et al. 2022). To compare drug resistance-associated genes (e.g. *rpoB, katG, ethA, inhA, pncA, embB, rrs, gyrA*, etc.; Fig. 7) across genomes, pyGenomeViz (https://github.com/moshi4/pyGenomeViz) was used to construct a synteny map. Developments are underway to make these tools accessible to the public. Alternatives also exist for interrogating a specific region of the genome, notably with tools such as GenBank (https://www.ncbi.nlm.nih.gov/genbank/). These analyses show high similarity (>99%) among genes, though their positions in genomes are unstable. Despite this, *M. tuberculosis* genomes exhibit high conservation. Integrating such analyses into pipelines and databases can provide global genomic insights and aid in deciphering drug resistance profiles.

## Conclusions and perspectives

In conclusion, molecular typing tools are crucial for studying TB epidemiology and evolution. Over the past three decades, various molecular methods have emerged, from IS*6110*-RFLP, once the gold standard, to more accessible techniques like spoligotyping and MIRU-VNTR typing, particularly in low-income countries. Currently, WGS is becoming the new gold standard for in-depth *M. tuberculosis* genome analysis. Despite this, no single typing system is ideal due to technical limitations and feasibility challenges, particularly in implementing WGS.

Recent advancements in TB-focused software and databases offer valuable insights into TB genomics, correlating data with geographical, demographic, and epidemiological information. However, further methods are needed to better analyze and extract meaningful knowledge from vast genomic data. Combining classical genotyping with WGS can provide a more comprehensive understanding of TB molecular epidemiology. Additionally, developing cost-effective, accessible WGS tools will help expand research in low-income settings. Future research will likely focus on leveraging AI to enhance the depth, accuracy, and efficiency of TB WGS data analysis.
